# Single-Wedge Lift-Out for Atom Probe Tomography Al/Ni Multilayers Specimen Preparation Based on Dual-Beam-FIB

**DOI:** 10.3390/mi13010035

**Published:** 2021-12-27

**Authors:** Yi Qiao, Yalong Zhao, Zheng Zhang, Binbin Liu, Fusheng Li, Huan Tong, Jintong Wu, Zhanqi Zhou, Zongwei Xu, Yue Zhang

**Affiliations:** 1 State Key Laboratory for Advanced Metals and Materials, University of Science and Technology, Beijing 100083, China; zhaoyalong0123@163.com (Y.Z.); bbliu@ustb.edu.cn (B.L.); 18810562702@163.com (F.L.); tonghuan416@163.com (H.T.); 2 School of Materials Science and Engineering, University of Science and Technology, Beijing 100083, China; zhangzheng@ustb.edu.cn; 3 State Key Laboratory of Precision Measuring Technology & Instruments, Laboratory of Micro/Nano Manufacturing Technology, Tianjin University, Tianjin 300072, China; 13352407489@163.com (J.W.); zzqtd@tju.edu.cn (Z.Z.)

**Keywords:** atom probe tomography (APT), single-wedge, lift-out, focused ion beam (FIB), Al/Ni multilayers

## Abstract

Atomic probe tomography (APT) samples with Al/Ni multilayer structure were successfully prepared by using a focused ion beam (FIB), combining with a field emission scanning electron microscope, with a new single-wedge lift-out method and a reduced amorphous damage layer of Ga ions implantation. The optimum vertex angle and preparation parameters of APT sample were discussed. The double interdiffusion relationship of the multilayer films was successfully observed by the local electrode APT, which laid a foundation for further study of the interface composition and crystal structure of the two-phase composites.

## 1. Introduction

Atomic probe tomography (APT) is the highest spatial resolution element analysis and testing equipment [1]. It mainly involves materials, physics, chemistry, biology and other research fields. The instrument is mainly used for atomic scale microanalysis in materials science research: in particular, measurement and analysis methods used in the research and development of materials. It provides researchers with a three-dimensional image of the material’s internal structure with atomic resolution. The sensitivity is close to one millionth. It is especially suitable for the study of nanoscale microstructure (precipitation, cluster, etc.), and various internal interfaces (grain boundary (GB), phase boundary, interlayer interface in multilayer structure, etc.) can be used to observe and study the segregation behavior, size and distribution of elements on the interface, and the distribution and composition of micro precipitates.

APT analysis has strict requirements on the shape of the sample: the shape of the sample is a symmetrical tip, the diameter of the tip top is less than 50 nm, the diameter of the tip bottom is less than 200 nm, and the effective analysis height is about 100–1000 nm. Although electrochemical methods are the dominant technique for preparing APT specimens [2,3], it is not easy for them to put the site-specific area (precipitation, cluster, GB, etc.) on the top of the APT needle specimens [4,5,6,7,8,9,10]. Scientists have taken decades years to figure out several basic tools for FIB-based specimen preparation used today for this challenge [11], known as the annular milling method [12,13], low-energy modification [14,15,16], site-specific lift-out method [17,18].

The FIB method is used to refine APT samples from top to bottom. When there is a huge phase structure difference between the two materials, the preparation of a multilayer structure is very difficult. At the same time, the Ga ion source for FIB cutting will induce an amorphous damage layer on the sample surface [19,20], while the resistance of the films to ion beam amorphous damage is weak. How to reduce or avoid the amorphous damage in the process of ion beam processing has become another difficulty in the preparation of APT multilayer composite materials.

Al/Ni multilayers are composed of alternating layers of Al and Ni, which have the characteristics of high chemical energy storage, fast energy release and fast combustion. They have potential application prospects in the following reaction ignition, thermal battery ignition and local heating. They are mainly used for welding and bonding [21]. A small thermal pulse will cause atoms to diffuse into the layer and lead to a rapid exothermic reaction, thus further establishing the self-propagating reaction [22]. In order to understand these behaviors, people have devoted themselves to basic research, such as final products, phase transition sequences and high concentration gradient effects. Particularly, the intermixing region in the interfaces not only changes the phase sequence, but also reduces stored chemical energy, especially as the modulation period drops below 50 nm. For instance, Al_9_Ni_2_ was observed as the primary phase in the multilayers with a modulation period larger than 25 nm, which could be explained by a nucleation model based on thermodynamics and diffusional intermixing. In this paper, Al/Ni multilayers APT sample were successfully prepared by the focused ion beam technique, which reduced the amorphous damage layer of Ga ions.

## 2. Experimental

Al/Ni multilayers with thickness of 4 μm were magnetron sputter-deposited from Al (99.999%) and Ni (99.999%) targets on a substrate of sapphire at a base pressure of 5 × 10^−4^ Pa. The modulation period was set to be 500 nm, while the relative thickness of Al and Ni layers was maintained at a 3:2 ratio. The temperature of the substrate was below 373 K during deposition. Both Al and Ni layer were deposited at 90 W, then the deposition rates for Al and Ni were about 15 nm/min and 10 nm/min, respectively.

Al/Ni multilayers were fixed on the FIB special sample preparation platform with silver glue. APT samples were prepared by Auriga focused ion beam field emission scanning double beam electron microscopy (FIB/SEM) produced by Zeiss (Oberkochen, Germany). APT samples were welded on the special Si column of APT by Pt welding, and the three-dimensional elements of APT samples were analyzed by the three-dimensional atomic probe system leap 5000 xr. The FIB system uses Ga ion as the ion source, the accelerating voltage is 3–30 kV, the working current is 50 pA to 20 nA, the minimum beam spot diameter is less than 3 nm; the acceleration voltage of the SEM system is 5 kV, and the image resolution is 1 nm.

## 3. Standard Lift-Out

**Figure 1:**
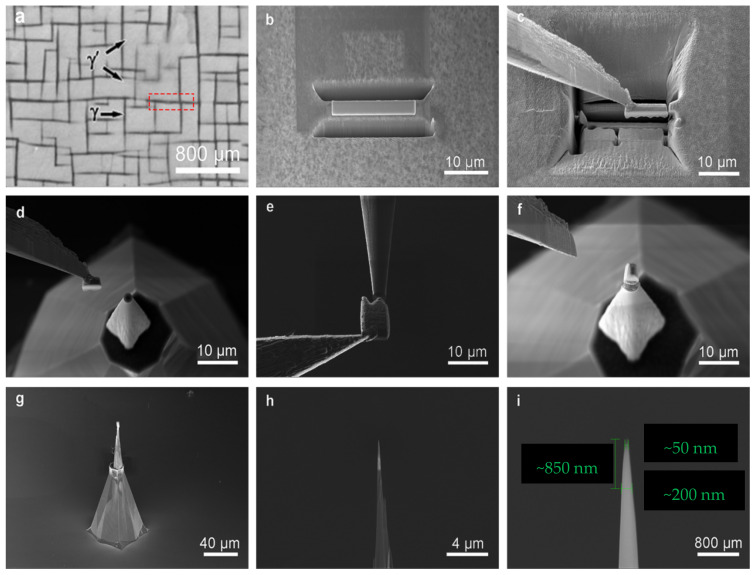
Example of the standard lift-out APT specimen preparation. (**a**) Platinum deposition; (**b**) cutting; (**c**) fine cutting; (**d**) lift-out; (**e**) welding; (**f**) separation; (**g**) annular milling; (**h**) cleaning; (**i**) fonfirmation.

When people are concerned about the three-dimensional distribution of special structural elements, such as the interface, precipitation, nanotubes and nanospheres, the typical lift-out method is usually used to prepare APT samples for analysis. This method was developed on the basis of Giannuzzi et al. (1997) [23], and Giannuzzi and Stevie (1999) [24], and then developed by Miller et al. (2005) [17], Miller and Russell (2007) [18], and Thompson et al. (2005–2007) [14,15,16]. The method is used as the main sampling method for region of interest (ROI). Taking the block interface material as an example, the sample preparation process of the standard method is shown in Figure 1: (a) platinum deposition, i.e., deposition of 20 μm × 2 μm × 1 μm platinum on the surface of the sample to protect the selected area of sample preparation; (b) cutting, that is, the thickness of the sample preparation area is reduced to 1 μm thickness by a small beam; (c) U-cut, that is, U-shaped separation between the thin sample and the matrix, but keeping a tiny connection; (d) lift-out, that is, thin samples are extracted from the matrix using nano-manipulators; (e) welding, that is, using Pt to weld the thin samples on an APT special holder; (f) separation, which is the separation of nano-manipulators and the sample; (g) annular milling, that is, sharpening the sample to a cone of 50–100 nm by annular beam flow; (h) cleaning, that is, cleaning the amorphous layer on the sample surface with a low voltage and low current ion beam; (i) confirmation, that is, the final shape of the sample should be tip configuration, with a tip top diameter less than 50 nm and a tip bottom diameter less than 200 nm. In this method, the parameters of centering during welding and the following annular milling/cleaning are the key to the success of the sample preparation.

## 4. Single-Wedge Lift-Out

### 4.1. Experimental Considerations

**Figure 2:**
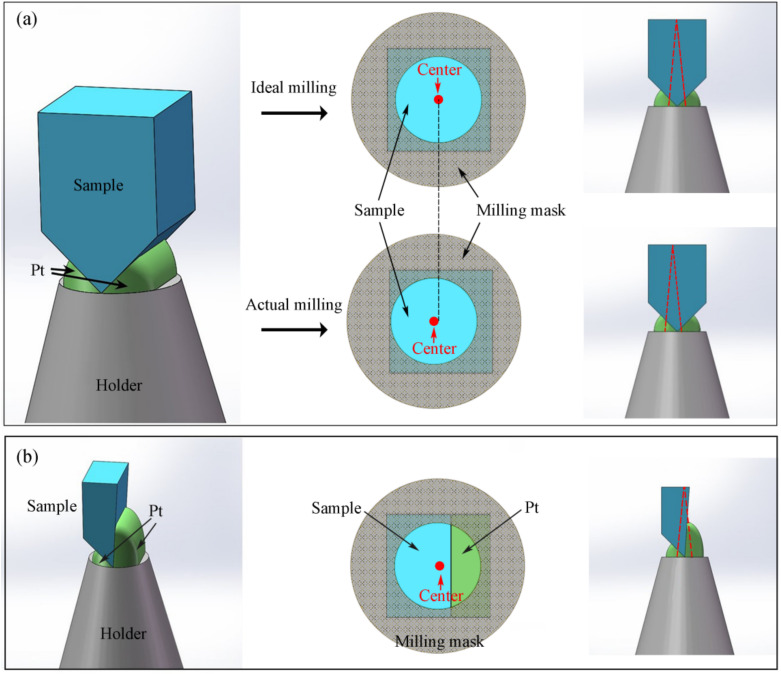
Welding process of standard lift-out and single-wedge lift-out. (**a**) Welding process of standard lift-out; (**b**) welding process of single-wedge lift-out.

**Figure 3:**
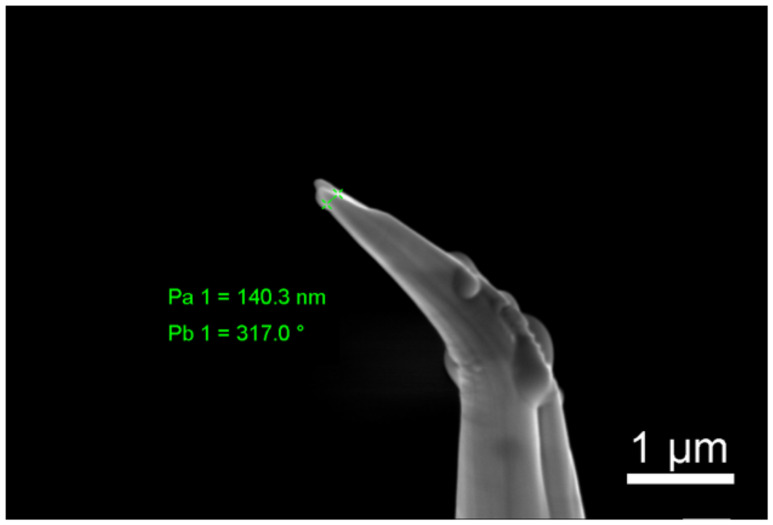
Example of the fractured APT sample.

In the standard lift-out method, the sample is proposed to be a wedge-shaped square column, as shown in Figure 2a. In the process of manual milling, the ion beam is perpendicular to the upper surface of the sample, and the area of the upper surface of the sample is larger than that of the lower surface of the sample, so it is difficult to determine the welding center. During the practical manufacture process, the welding center of the sample is easy to produce deviation, thus cutting out the PT for welding, as shown in Figure 2a, which makes welding the joint very fragile, resulting in the sample fracture in APT analysis as shown in Figure 3. Therefore, we hope to find a suitable sample shape so that the solder joint between the sample and the holder can be easily determined during manual milling so as to strengthen the welding. Our new design strategy is to change the wedge lift sample into a single wedge, as shown in Figure 2b. In this way, when the ion beam is observed, the welding point between the sample and the bracket can be easily obtained, and enough welding platinum can be reserved during the encircling process to strengthen the welding stiffness of the sample.

### 4.2. Single-Wedge Lift-Out Tip Fabrication

#### 4.2.1. Single-Wedge Milling

In the standard lifting method, we use Auriga fiber reinforced plastics produced by Zeiss. When the tilt angle of the sample stage is 54°, the ion beam is perpendicular to the upper surface of the sample. When milling, the tilt angle of the sample table is 54 ± 2° and the bottom of the double wedge specimen is thin, which can shorten the U-shaped cutting time and avoid the phenomenon of re deposition welding. In the single wedge ejection method, considering that the focusing plane of the ion beam is the upper surface of the sample, there is defocusing phenomenon from the ion beam to the bottom of the sample. In order to eliminate the defocusing effect and obtain the vertical plane, the tilt angle of the sample table is 55° during milling. At the same time, in order to keep the advantage of easy separation between the standard lift-out sample and the matrix during U-shaped cutting, it is necessary to increase the milling angle of the wedge side to 51°.

#### 4.2.2. Off-Center Welding

**Figure 4:**
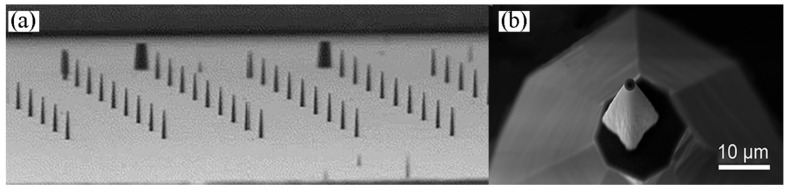
APT Si holder. (**a**) APT Si holders; (**b**) single APT Si holder.

**Figure 5:**
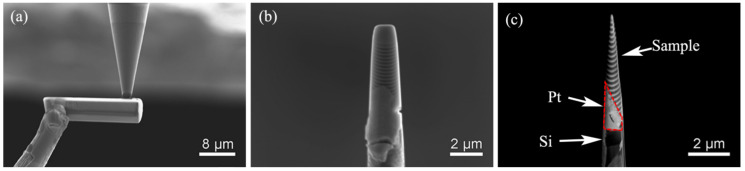
Welding process of single-wedge lift-out. (**a**) Welding; (**b**) sample enlarged view; (**c**) sample fine profile.

The APT sample holder we used is the APT special holder of CAMECA Company. It is a Si conical cylinder with a 2 μm upper surface, as shown in Figure 4. In the standard lifting-out method, the wedge tip of the sample is welded to the center of the upper surface of the silicon column to facilitate alignment, while in the single-wedge-ejection method, the wedge tip of the sample is welded at two thirds of the upper surface of the silicon column, with the vertical plane inside and the wedge body outside, as shown in Figure 5.

#### 4.2.3. Annular Milling

**Figure 6:**
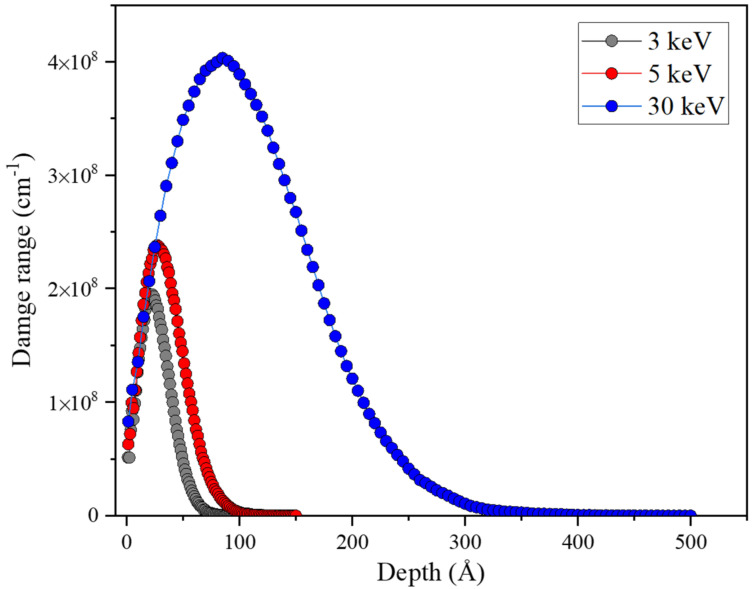
SRIM results for Ga ion induced recoil damage distribution in Al/Ni substrate under different ion energies.

**Figure 7:**
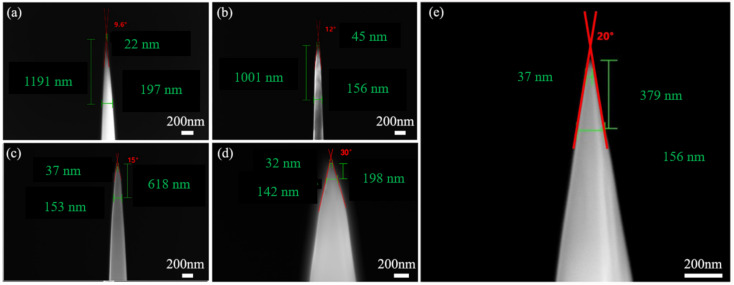
Example of the single-wedge lift-out APT specimen tip shape. (**a**) Tip 9°; (**b**) tip 12°; (**c**) tip 15°; (**d**) tip 30°; (**e**) tip 20°.

SRIM-2013 is used to qualitatively analyze the Ga ion induced damage distribution in Al/Ni substrate from the perspective of Monte Carlo simulation, as shown in Figure 6. Among them, the Ga ion induced damage peaks are at ~2.2 nm, ~3.1 nm and ~8.5 nm, corresponding to 3 keV, 5 keV and 30 keV, respectively. It can be seen that the damage peaks ranging from 2 nm to above 10 nm with an impact energy ranges from 3 keV to 30 keV, showing a significantly increasing trend of damage concentration. In experimental studies, the tip shape is also an important factor in APT analysis. It is well known that there are many defects, such as vacancies, at the interface between the grain boundary and the phase boundary in the three-dimensional atomic probe experiment, which can lead to the fracture of the interface tip. As shown in Figure 7, we take the uniform silicon sample as the research object, and improve the stability of the sample by controlling the shape of the tip so as to avoid the tip fracture at the interface. The results show that when the taper is greater than 12° and in order to consider the sample collection amount of APT, the optimal cone angle of the tip is about 20° as shown in Figure 7e. In order to obtain the taper angle, it is necessary to control the diameter D2 and D1 of the grinding mask. After the inner ring is determined, if the outer ring diameter is reduced, the cutting area will be greatly reduced, and the cutting efficiency will be improved, but the needle tip will also become steep and vulnerable to brittle fracture; if the outer ring diameter is larger, the cutting area will be larger, and the cutting efficiency will be reduced, but the cone angle of the needle tip will be significantly larger, and there will be burr on the outside of the needle tip, which will affect the APT analysis results.

**Figure 8:**
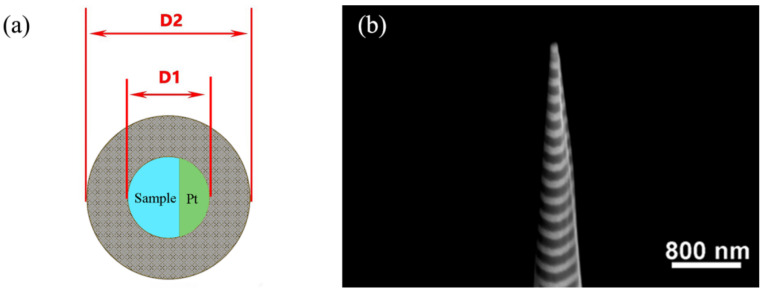
Example of annular milling and the APT specimen tip shape of the Al/Ni multilayers. (**a**) Schematic diagram of annular milling; (**b**) the APT specimen tip shape of the Al/Ni multilayers.

**Figure 9:**
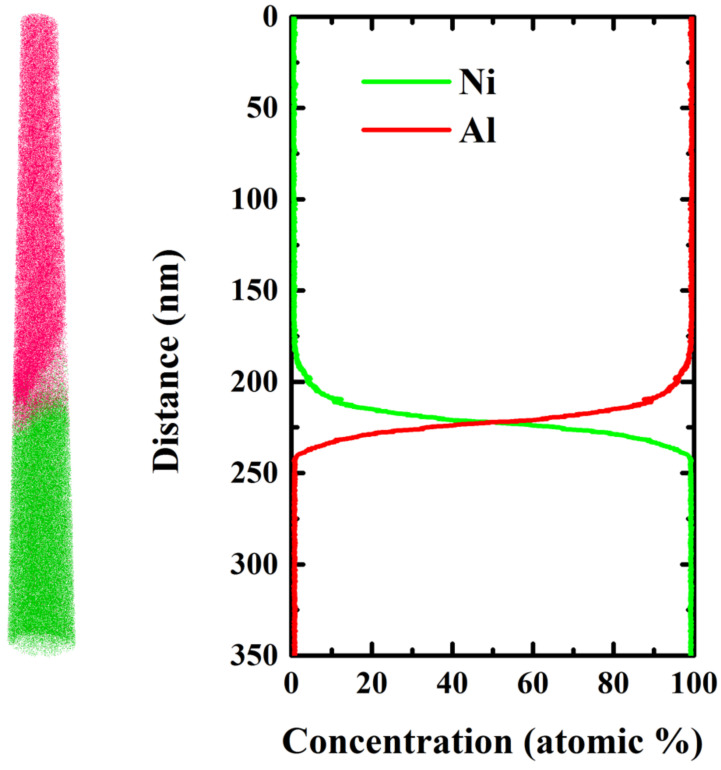
Reconstruction of atomic distribution and concentration profiles of the as-deposited Al/Ni multilayers.

When the cutting current of the Ga ion beam is about 30 kV, the amorphous layer is about 20–30 nm. When the cutting beam voltage drops to 3 kV and 5 kV, the thickness of the amorphous layer can be reduced to 1–5 nm. Therefore, in order to reduce the thickness of the amorphous layer of Al/Ni multilayers, the hierarchical voltage dilution method is used in the manual milling process, as shown in Table 1. Finally, the diluted sample is cleaned with 5 kV and 3 kV low pressure for 2–3 min, and the cleaning effect is good, as shown in Figure 8. The results of the APT analysis are shown in Figure 9.

## 5. Conclusions

In this paper, the nanofabrication technology of APT sample preparation of biphasic composites was studied by focused ion beam field emission scanning electron microscope, by improving the lift-out method with eccentric welding, tip angle control and step voltage thinning. The visualization of the welding between the wedge bottom and the holder was realized, so that the wedge and the holder were welded firmly, and the sample was on the back side, so it was not easy to fracture in the preparation of manual milling and APT analysis. The optimum vertex angle and preparation parameters of APT sample were discussed. APT samples with the Al/Ni multilayer structure with the reduced amorphous damage layer of Ga ions were successfully prepared. It is of great significance to study the interface composition, crystal structure and diffusion mechanism of Al/Ni dual phase composites.

## Figures and Tables

**Table 1:** The sample diameter versus FIB process parameter.

Tip Diameter D/nm	Ion Beam Voltage U/kV	Ion Beam Current I/pA
1000	30	240
200	15	120
100	10	20
50	10	2
